# Transcriptomic Response to Nitric Oxide Treatment in *Larix olgensis* Henry

**DOI:** 10.3390/ijms161226117

**Published:** 2015-12-02

**Authors:** Xiaoqing Hu, Jingli Yang, Chenghao Li

**Affiliations:** State Key Laboratory of Tree Genetics and Breeding, Northeast Forestry University, 26 Hexing Road, Harbin 150040, China; 15246344643@163.com (X.H.); yifan85831647@sina.com (J.Y.)

**Keywords:** *Larix olgensis* Henry, transcriptome, nitric oxide, disease resistance

## Abstract

*Larix olgensis* Henry is an important coniferous species found in plantation forests in northeastern China, but it is vulnerable to pathogens. Nitric oxide (NO) is an important molecule involved in plant resistance to pathogens. To study the regulatory role of NO at the transcriptional level, we characterized the transcriptomic response of *L. olgensis* seedlings to sodium nitroprusside (SNP, NO donor) using Illumina sequencing and *de novo* transcriptome assembly. A significant number of putative metabolic pathways and functions associated with the unique sequences were identified. Genes related to plant pathogen infection (*FLS2*, *WRKY33*, *MAPKKK*, and *PR1*) were upregulated with SNP treatment. This report describes the potential contribution of NO to disease resistance in *L. olgensis* as induced by biotic stress. Our results provide a substantial contribution to the genomic and transcriptomic resources for *L. olgensis*, as well as expanding our understanding of the involvement of NO in defense responses at the transcriptional level.

## 1. Introduction

Nitric oxide (NO) is a redox-active reactive nitrogen species and an important endogenous signaling molecule in plants. NO appears to be involved in various plant developmental and physiological processes such as stomatal closure, root growth, disease resistance, and abiotic stress resistance [1,2,3]. In plants, NO production is facilitated by nitrate reductase (NR) as well as nitric oxide synthase (NOS) pathways [4]. In addition, NO can also be generated from nonenzymatic sources [5]. In fact, upregulated expression of NO synthase (NOS) in *Arabidopsis* increases abiotic and biotic stress tolerance [6]. It was reported that ectopic expression of NOS genes in tobacco results in enhanced biotic stress resistance [7]. Thousands of NO-responsive genes have been identified previously, most of which are stress-related, and function in plant defense [8,9,10,11]. However, the NO regulatory mechanisms of woody plants in response to biotic stress are poorly understood.

Next-generation sequencing (NGS) technologies are low-cost, high-throughput sequencing methods that can rapidly generate information on a large number of genes. Transcriptome sequencing is a convenient and effective means of gene discovery [12,13], especially with the use of high-throughput NGS technologies. Transcriptome sequencing provides a wide range of genome information that can facilitate novel gene discovery and transcription factor (TF) discovery [14,15,16].

Olga Bay larch (*Larix olgensis* Henry), which is widely distributed in the Changbai Mountain region of Northeast China, North Korea and eastern Russia, is a common temperate, coniferous tree species in East Asia [17]. This species is one of the most ancient larches and is listed in the Red Book of the Russian Federation [18]. It is also one of the most industrially important coniferous species in the plantation forests in northeastern China because its wood products have anti-corrosive properties. China holds the largest total plantation area of *L. olgensis* for conifers. Unfortunately, *L. olgensis* is easily ravaged by a number of pathogenic fungi such as *Botryosphaeria laricina* (Sawada) Shang and *Cladosporium tenuissimum* Cook, leading to substantial losses of forests. To date, the transcriptomes of multiple larch species, including *L. kaempferi* × *L. olgensis* [19], *L. gmelinii* [20], and *L. leptolepis* [21] were sequenced by NGS. Unfortunately, information on the *L. olgensis* genomic sequence is limited, and only a few regulatory genes for biotic stress resistance have been identified.

To investigate the molecular mechanism of NO in *L. olgensis*, sequencing and *de novo* transcriptome assembly of seedlings pretreated with sodium nitroprusside (SNP, NO donor) was performed in the present study. Our results indicate that NO plays important roles in various processes, such as significant changes in the expression of genes involved in biotic stress resistance and cell wall biosynthesis. This study explores the possible mechanisms of disease-resistance at the transcriptional level, as well as provides a substantial contribution to the genomic and transcriptomic resources of *L. olgensis*.

## 2. Results and Discussion

### 2.1. NO Production in Response to Sodium Nitroprusside (SNP)

*L. olgensis* seedlings were irrigated in a various concentrations of SNP. Compared with the control sample, there were no observable changes to seedlings treated with 33.5 and 167.8 μM SNP; however, there was visible wilting on seedlings treated with 839 μM SNP.

**Figure 1:**
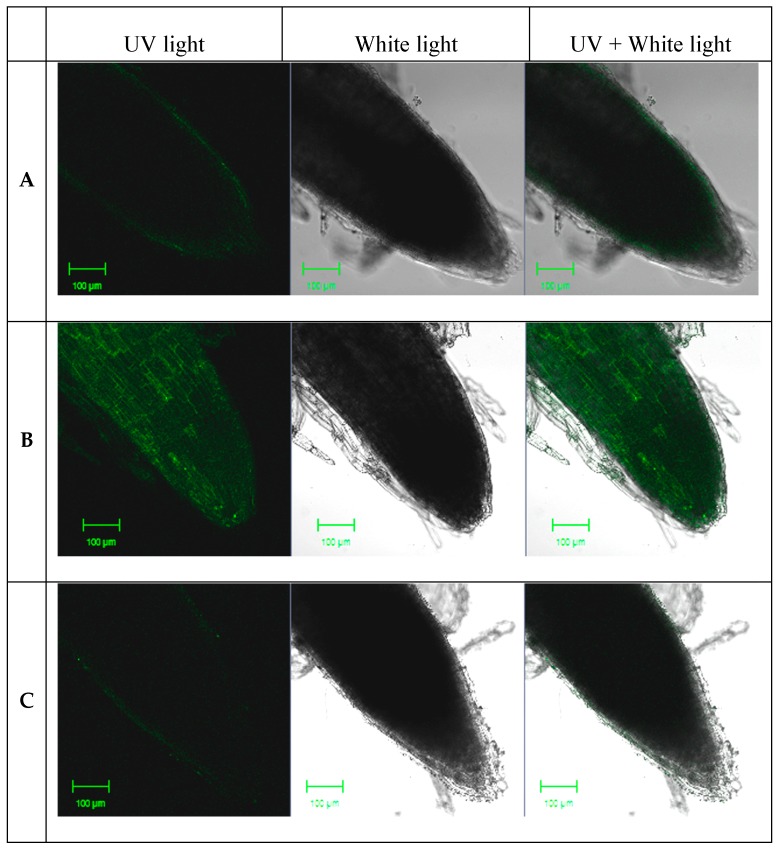
Effect of SNP on nitric oxide (NO) generation in *L. olgensis* seedlings. NO fluorescence was detected using the NO-specific 4-amino-5-methylamino-2′,7′-difluorofluorescein diacetate (DAF-FM-DA) green fluorescent dye, and viewed by confocal laser scanning microscopy. (**A**) Root of control seedlings; (**B**) Root of seedlings treated with 167.8 μM sodium nitroprusside (SNP, NO donor) for 5 h; (**C**) Root of seedlings treated with 167.8 μM SNP and 100 μM 2-4-carboxyphenyl)-4,4,5,5-tetramethyl-imidazoline-1-oxyl-3-oxide (cPTIO) for 5 h.

NO was visualized using the specific fluorescent probe 4-amino-5-methylamino-2′,7′-difluorofluorescein diacetate (DAF-FM-DA) to detect NO fluorescence. Fluorescence analysis revealed that the control seedlings (Figure 1A) and seedlings treated with 33.5 μM SNP displayed weak fluorescence, whereas those treated with 167.8 μM SNP for 5 h showed a significant increase in fluorescence intensity (Figure 1B). When incubated with 2-(4-carboxyphenyl)-4,4,5,5-tetramethyl-imidazoline-1-oxyl-3-oxide (cPTIO), fluorescence intensity markedly decreased (Figure 1C). Based on these results, the seedlings treated with 167.8 μM SNP for 5 h were selected for use in subsequent RNA expression analyses.

### 2.2. Functional Annotation of Unigenes

**Figure 2:**
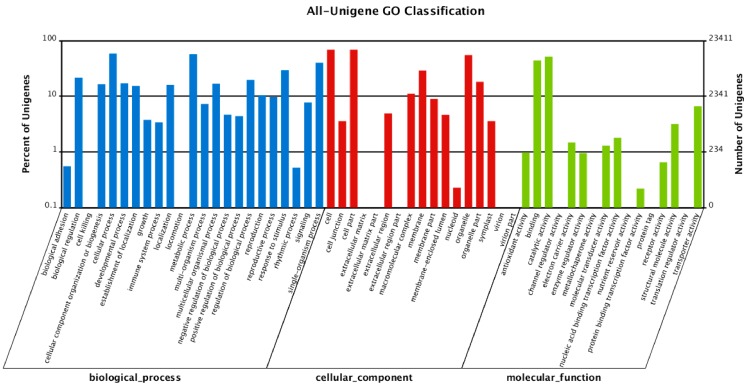
Gene ontology (GO) classifications. GO function is showed in *X*-axis. The right *Y*-axis indicates the number of genes with GO functions, and the left *Y*-axis indicates the percentage in a logarithmic scale.

To explore the regulatory role of NO in *L. olgensis*, sequencing and *de novo* transcriptome assembly were performed. Unigenes were annotated with the Nr, NT, Swiss-Prot, KEGG, COG, and GO databases. To identify unique sequences, similarity searches were performed against the NCBI Nr database using BLASTx (http://www.ncbi.nlm.nih.gov). Of the mapped unigenes, 53.5% and 32.8% had significant homology (≤80%) and an *E*-value less than 1 × 10^−5^ (Figure S1A,B). Searches against the COG database led to 26,012 unigenes being divided into 25 categories (Table S1, Figure S2). The largest number of unigenes were predicted to have general functions (4188 unigenes; 16.1%), followed by transcription (2141 unigenes; 8.2%). A total of 175,387 unigenes were classified into 57 GO annotations, which belong to one of the three GO categories: molecular function (26,402), cellular components (64,721) or biological processes (84,264) (Figure 2, Table S2). The largest number of unigenes (16,067 unigenes, 24.8%) in a GO annotation was observed in the cellular components category (Figure 2).

### 2.3. Investigation of Differentially Expressed Transcripts under SNP Treatment

Exposure of *L. olgensis* roots to 167.8 μM SNP resulted in the upregulation of 2671 genes and downregulation of 3674 genes (Figure S3). We analyzed the 50 most differentially expressed genes (DEGs) between control and SNP treatments and identified nine upregulated and one downregulated gene (Table S3) that were involved in various important biological pathways, including cyanoamino acid metabolism, tryptophan metabolism, aminobenzoate degradation, styrene degradation, and nitrogen metabolism (Table 1). It is likely that these highly expressed genes may play important roles in biological functions, and need to be investigated further in future studies.

**Figure 3:**
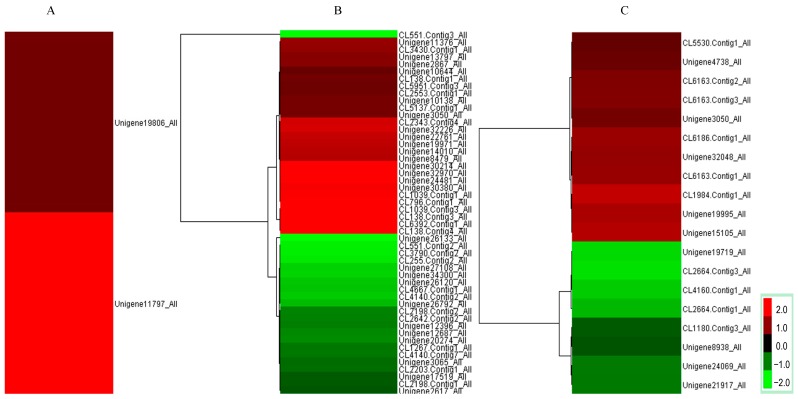
Clustering of differentially expressed genes. Red colors indicate up-regulation, and green colors indicate down-regulation. (**A**) Differentially expressed genes related to nitrate reductase activity; (**B**) Differentially expressed genes related to the response to organic nitrogen; (**C**) Differentially expressed genes related to nitrate transport.

For nitrogen metabolism, several DEGs (Table S4) were determined to be involved in nitrate reductase activity (Figure 3A), nitrate transport (Figure 3B), and response to organic nitrogen (Figure 3C). Two NR genes were transcriptionally induced by NO treatment (Figure 3A), which was similar to previous findings involving birch cells in suspension [22]. There is growing evidence that NR is one of the main potential sources of endogenous NO in plants [4]. Therefore, our results support previous findings that exogenous NO is involved in the regulation of endogenous NO signals in larch seedlings.

Among the genes with KEGG pathway annotations, 2670 DEGs were identified between the control and SNP libraries. Pathway enrichment analysis showed that 120 pathways were significantly enriched (Q value ≤ 0.05) in DEGs detected between the control and SNP libraries (Table S5). Most DEGs were associated with metabolic pathways (945 members), biosynthesis of secondary metabolites (573 members), ribosome (213 members), and phenylpropanoid biosynthesis (200 members).

**Table 1:** Gene pathways enriched in the top 50 highly expressed genes.

Unigene	Pathways (Control *vs.* SNP)	Nr-ID
Unigene32649_All	Cyanoamino acid metabolism, Tryptophan metabolism, aminobenzoate degradation, styrene degradation, Nitrogen metabolism	gi|348690846|gb|EGZ30660.1|
Unigene32533_All	Carbon metabolism, biosynthesis of amino acids, glycolysis/gluconeogenesis, carbon fixation in photosynthetic organisms, HIF-1 signaling pathway, Alzheimer‘s disease	gi|226495473|ref|NP_001147336.1|
CL6269.Contig1_All	RNA transport, Legionellosis	gi|301111276|ref|XP_002904717.1|
Unigene32730_All	Ribosome	gi|323451608|gb|EGB07485.1|
CL5964.Contig2_All	Mineral absorption	gi|357161316|ref|XP_003579051.1|
Unigene32753_All	Spliceosome	gi|145324176|ref|NP_001077677.1|
Unigene26640_All	Plant-pathogen interaction	gi|2224913|gb|AAB61709.1|

### 2.4. Protection against Pathogen Infection

**Figure 4:**
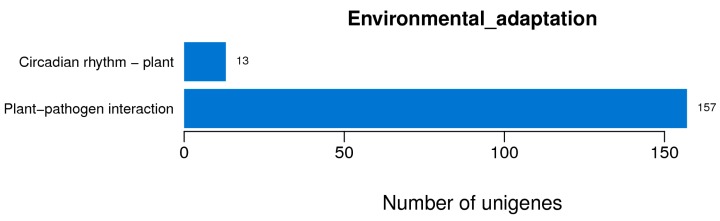
Enrichment of differentially expressed genes (DEGs) related to environmental adaptation. The number of DEGs annotated in this pathway is indicated.

Plants are constantly exposed to a range of pathogenic microbes, and possess elaborate defense mechanisms to prevent infection. Our transcriptome analysis result revealed that many genes (156 members) in the plant-pathogen interaction category were differentially expressed between control and NO-treated plants (Figure 4, Table S6).

Innate immunity in plants is first triggered by the perception of pathogen-associated molecular patterns (PAMPs) in the presence of surface-localized pattern recognition receptors (PRRs) [23,24]. In *Arabidopsis*, the leucine-rich repeat receptor-like kinase (LRRRLK) flagellin-sensing2 (FLS2) binds to the bacterial PAMP flagellin, initiating PAMP-triggered immunity. The EF-Tu receptor (EFR) has also been extensively characterized. It recognizes the bacterial elongation factor Tu (EF-Tu). The Arabidopsis PAMP chitin, a component of the fungal cell wall, which is recognized by the LysM-RLK chitin elicitor receptor kinase1 (CERK1), also induces a defense response. In the present study, several genes encoding PRRs were differently expressed with NO treatment, including 37 FLS2 genes (16 upregulated and 21 downregulated), eight EFR genes (all down regulated), and one CERK1 gene (downregulated) (Table S6). This is the first report that shows that NO induces the differential expression of PRR genes. Genome-wide transcriptional response of plants to NO treatment has been reported in *Arabidopsis* [10,25], birch cells, and other plants; however, studies showing that NO alters the expression of *PRR* genes are limited [22]. NO treatment significantly increased the expression of some FLS2 genes in this study (which included 4 genes out of the 20 most upregulated). The present study has not determined whether FLS2 acts downstream of NO to reverse the effects of PAMPs. However, direct evidence suggests that NO may have a close relationship with PAMPs and PRR in plant defense.

Another important finding of the present study was that mitogen-activated protein kinase kinase kinase (MAPKKKs) were induced by NO at transcriptional level. This result was consistent with results reported for *Arabidopsis* and tobacco, indicating that NO upregulates mitogen-activated protein kinases (MAPKs) [6,7]. MAPKs are organized into signaling cascades that form the backbone of the signaling network within and between cells [26,27]. Several MAPKs have been demonstrated to be involved in the plant response to biotic stress [28,29,30]. These include the upstream receptors, MAPKKs, and MAPKKKs. MAPK-dependent and MAPK-independent signaling pathways act downstream of FLS2 to activate the *WRKY* gene [31]. This signaling responds to both fungal and bacterial pathogens and could be engineered to enhance disease resistance [31].

An important transcriptional regulator of defense gene *Pathogenesis-related protein 1* (*PR1*), was dramatically upregulated with SNP treatment (Table S6) [32]. Previous reports in *Arabidopsis* have shown that NO treatments increase *PR1* expression [11]. In defensive signaling processes of plants, NO is an important component in the hypersensitive response (HR), which occurred when potential pathogens are trapped near the site of infection [33]. In this process, NO activates guanylate cyclase, thereby initiating cGMP-mediated signal transduction [33], as well as protein modifications by *S*-nitrosylation [34]. These subsequently regulate the transcription of *PR1*. NO also regulates an NADPH oxidase and thereby controls HR-associated cell death using the same process [35].

In addition, six disease resistance protein genes were upregulated by SNP treatment (Table S6). These include four genes encoding resistance to *Pseudomonas syringae 2* (*RPS2*), one gene encoding resistance to *Pseudomonas syringae 5* (*RPS5*), and one gene encoding resistance to *P. syringae* pv *maculicola 1* (*RPM1*). Overexpression of RPS2 [36], RPS5 [37], or RPM1 [38] induces disease resistance, suggesting that the responses of larch seedlings to SNP resemble biotic stress responses.

NO also upregulated some transcription factors that are involved in inducing plant defense responses. Of particular interest are the WRKY transcription factors (Table S6). Previous reports involving *Arabidopsis* have shown that NO treatments increase expression of *WRKY* genes [10]. The transcription of *WRKY* genes is strongly and rapidly upregulated in response to wounding in numerous plant species, pathogen infection, or abiotic stresses [39,40,41]. Several studies suggested the function of WRKYs is to bind to the W box element (TTGACC/T) [42,43], which is located in the promoter regions of various plant defense genes [44,45]. An approximate two-fold induction of *WRKY22* and *WRKY33* was observed with SNP treatment (Table S6). Previous studies have shown that the overexpression of *AtWRKY33* enhances resistance to necrotrophic fungal pathogens *Botrytis cinerea* and *Alternaria brassicicola* [46]. In addition, *AtWRKY33* is reported as a positive regulator of pathogen-induced autophagy, an important process for plant resistance to pathogenic necrotrophic fungi [47].

### 2.5. Cell Wall Biosynthesis

The phenylpropanoid pathway is an important process in the synthesis of defensive compounds [48]. Lignin is one of the products of the phenylpropanoid pathway. It is reported that lignin can be used as a physical barrier against initial pathogen colonization [49]. Also, the lignin deposited in infected cells prevents the spread of pathogenic toxins and enzymes, as well as inhibits water and nutrient transport from the plant cells to the pathogen [50]. In addition, some genes involved in lignin biosynthesis are induced, and lignins are deposited rapidly in secondary cell walls [51]. Transcriptional profiling of various plant-pathogen interactions have also revealed the activation of genes whose products are involved in the modification of cell wall components [52,53].

**Figure 5:**
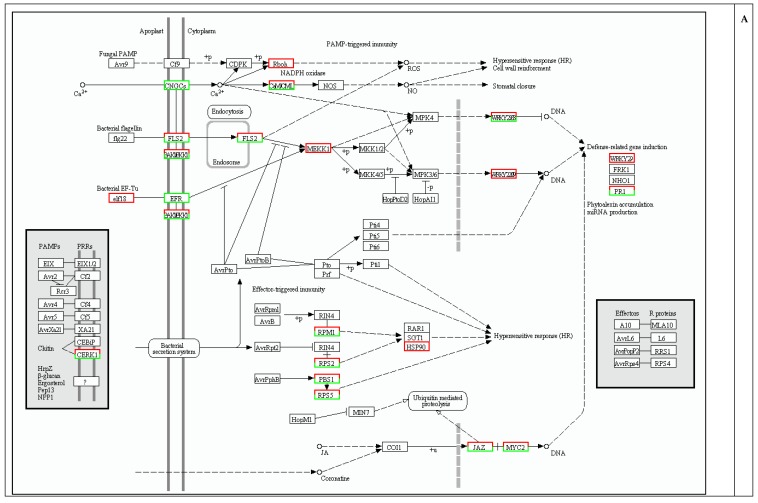

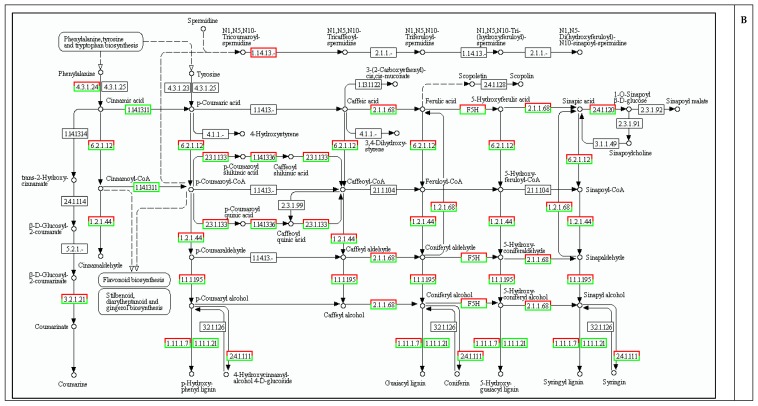
Plant-pathogen interactions and phenylpropanoid biosynthesis pathways induced by sodium nitroprusside treatment (SNP, NO donor). Black borders: Pathway; Red represents up-regulated genes; Green represents up-regulated genes. (**A**) Plant-pathogen interaction; (**B**) Phenylpropanoid biosynthesis.

A total of 97 DEGs involved in the phenylpropanoid biosynthesis pathway were identified in the present study (Figure 5B). Several DEGs were related to lignin synthesis, including genes coding for 4-coumarate CoA ligase (4CL), caffeoyl-CoA *O*-methyltransferase (CCoAOMT), cinnamoyl-CoA reductase (CCR), cinnamyl alcohol dehydrogenase (CAD), phenylalanine ammonia-lyase (PAL), cinnamate-4-hydroxylase (C4H), ferulate-5-hydroxylase (F5H), and peroxidase (POD). These proteins are involved in the metabolic activity of higher plants and are key enzymes for the monolignol biosynthesis pathway. This pathway is also involved in Scots pine defense against root rot disease [53]. Similarly, in silk spruce, seven DEGs related to the phenylpropanoid biosynthesis pathway of *P. sitchensis* phloem are involved in plant defense responses to wounding or insects [54]. Some DEGs are involved in the phenylpropanoid pathway after treatment with NO, which includes *C4H*, *PAL*, and *CAD* [10,11], and this is supported by the results of the present study. In addition, differentiating xylem cells of young *zinnia* seedlings showed outbreaks of NO production at an early stage of the differentiation process, which were sustained during cell wall synthesis [55]. Agnieszka [56] found out that NO was generated in differentiating vessels of roots in *P. trichocarpa*, through the beginning of the differentiation process. The locations of NO accumulation co-localized to the sites of cell wall thickening. This finding is significant because it indicates that lignin biosynthesis might be induced by NO which activates the enzymes related to this pathway. Moreover, a previous study clearly showed that NO is produced in lignin biosynthesis when plants are exposed to wounding [57]. When NO was eliminated by cPTIO, cell wall lignification was clearly reduced [57]. Based on the above results, we speculate that the changes in the expression of genes involved in cell wall biosynthesis after NO treatment result in cell wall thickening and increased disease resistance, although further verification is warranted.

### 2.6. Validation of Transcriptomic Data by QRT-PCR

**Figure 6:**
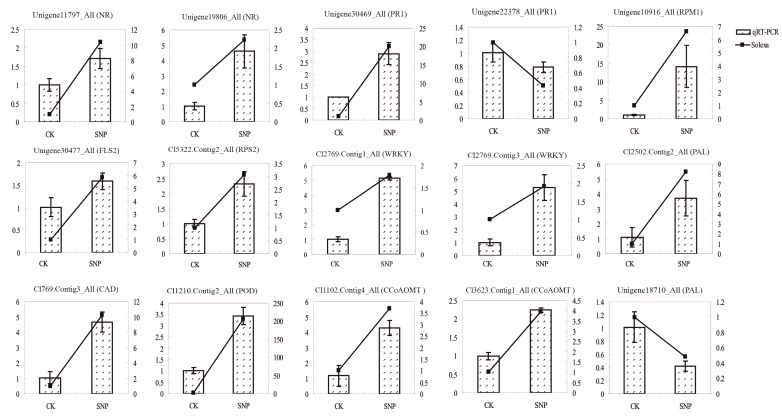
QRT-PCR analysis of selected genes. Fifteen genes were selected to verify the transcriptomic data. The histogram represents qRT-PCR analysis and the line chart represents RPKM (Reads per Kilobase of exon model per Million mapped reads) values. The *X*-axis stands for different samples. The right *Y*-axis stands for relative expression level and the left *Y*-axis indicates RPKM values. Standard deviations were derived from three replicates of each experiment.

Two genes annotated to nitrate reductase were highly expressed (unigene11797_All, unigene19806_All) (Figure 6). Six genes related to plant pathogen infection were also validated: unigene30469_All and unigene22378_All annotated as PR1; Unigene10916_All, unigene30477_All and cl5322.Contig2_All identified as the disease resistance proteins RPM1, FLS2 and RPS2, respectively (Figure 6). Both cl2769.Contig1_All and cl2769.Contig3_All were named as WRKY. Genes involve in the phenylpropanoid pathway such as *PAL* (unigene18710_All), *CAD* (cl769.Contig3_All), *POD* (cl1210.Contig2_All) and *CCoAOMT* (cl3623.Contig1_All, cl1102.Contig4_All) were also up-regulated, while another *PAL* gene (cl2502.Contig2_All) was down-regulated. Thus, the results of qRT-PCR consistently matched with the initial transcriptome analysis, and therefore support the results.

## 3. Experimental Section

### 3.1. Plant Materials and Treatments

Open-pollinated mature seeds of *L. olgensis* were collected during mid-September from Qingshan Forestry Bureau Seed Orchard, Heilongjiang Province, China, and were stored in sealed plastic bags at −20 °C. The seeds were sown on the surface of a mixture of equal parts soil and vermiculite for germination.

Six-week-old of seedlings were rinsed in running tap water to remove surface exudates, and irrigated with 33.5, 167.8, or 839 μM SNP in distilled water for 5 h. Seedlings treated with distilled water were used as control. For scavenger treatment, seedlings were irrigated with 100 μM cPTIO and 167.8 μM SNP for 5 h. After treatment, the seedlings were incubated in Mes/KCl buffer (5 mM KCl, 10 mM Mes, 50 M CaCl_2_, pH 6.15) for 2 h, and then in 10 μM of the NO indicator fluorescent dye DAF-FM-DA (Sigma-Aldrich, St. Louis, MO, USA) for 10 min, washing with Mes/KCl buffer for 20 min. Fluorescence was visualized at 488 nm using a confocal laser scanning microscope (Carl Zeiss LSM700, Jena, Germany).

### 3.2. RNA Extraction, cDNA Library Preparation, and Illumina Sequencing

Six-week-old seedlings were soaked in 167.8 μM sodium nitroprusside (SNP) or sterile distilled water (control) for 5 h. Ten individual seedlings were collected and pooled into one sample for sequencing. Total RNA was extracted using a modified CTAB method [58] and digested with RNase-free DNase I (Promega, Madison, WI, USA) at 37 °C for 30 min. Both the concentration and integrity of the RNA samples were evaluated using a NanoPhotometer (GmbH, Munich, Germany). MRNA was isolated from total RNA using Dynabeads oligo (dT) (Invitrogen, Carlsbad, CA, USA). First-strand cDNA was generated using random hexamer primers. The second-strand cDNA was generated using buffer, dNTPs, RNaseH, and DNA polymerase I (Invitrogen). Double-stranded cDNA was used for library construction. The short PCR fragments were purified with a QiaQuick PCR extraction kit, and suitable fragments, here meaning fragments that had sequencing adaptors, were selected as templates for PCR amplification based on the results of agarose gel electrophoresis. For quality control an Agilent 2100 Bioanalyzer (Agilent Technologies, Inc., Santa Clara, CA, USA) and ABI StepOnePlus™ Real-Time PCR System (Applied Biosystems, Foster City, CA, USA) were used for quantification and to evaluate library quality. Libraries were then sequenced on an Illumina HiSeq™ 2000. The raw Illumina data have been deposited in SRA at NCBI with accession numbers (control: SAMN02665256; SNP: SAMN02665243).

### 3.3. De Novo Assembly and Annotation

By removing empty reads, adaptor sequences, and low-quality sequences to obtain the clean reads. Then assembled the clean reads into contigs using Trinity [59], and were then connected using Unigene (Table 2). When multiple samples from the same species are sequenced, Unigenes from each sample’s assembly can be analyzed further to identify splicing and to remove redundancy with sequence clustering software. Next, BlastX alignments [60] (*E*-value < 10^−5^) were performed between Unigenes and the following protein databases: NCBI non-redundant protein (Nr) (http://www.ncbi.nlm.nih.gov), Swiss-Prot [61], KEGG [62], and COG [63]. The highest sequence similarity to a gene in the NCBI Nr database was annotated to the Unigene. Gene Ontology (GO) annotations for the unigenes were determined using Blast2GO [64], and the WEGO [65] was used to analyze GO functional classifications.

**Table 2:** Details of the sequencing and assembly.

Parameters	Control	Sodium Nitroprusside
Total number of reads	51,426,920	54,580,304
Total nucleotides (bp)	4,628,422,800	4,912,227,360
GC percentage	46.11%	46.54%
Q20 percentage	98.15%	98.28%
Total number of contigs	101,853	95,211
Length of all contigs (bp)	39,753,573	38,513,317
Contigs N50 (bp)	872	910
Mean length (bp) of contigs	390	405
Total number of unigenes	65,207	58,080
Length of all unigenes (bp)	38,628,763	38,445,887
Mean length (bp) of contigs	592	662
Unigenes N50 (bp)	958	1117

### 3.4. qRT-PCR Validation

Total RNA was extracted from SNP-treated and non-treated seedlings of *L. olgensis* Henry plants using method mentioned previously. First-strand cDNA was synthesized with 0.5 μg purified RNA and reverse-transcribed with a Reverse Transcriptase kit (TaKaRa Biotech, Dalian, China). qRT-PCR reactions were performed in a volume of 20 μL, containing 10 μL of SYBR premix ExTaq (TaKaRa Biotech, Dalian, China), 0.5 μM of forward and reverse primers, and 2 μL cDNA template (equivalent to 0.05 μg of total RNA). Thermal cycling conditions were performed as follows: 95 °C for 10 s, followed by 40 cycles of 95 °C for 5 s, 60 °C for 30 s, and 78 °C for 1 s for plate reading. The primer sequences used for α-tubulin and 15 selected genes related to NR, plant-pathogen interactions, and the phenylpropanoid pathway that were used to validate the RNA-seq data are presented in Table S7.

## 4. Conclusions

The present study examined the potential contribution of NO to disease resistance in *L. olgensis* by analyzing the effect of NO treatment on defense-related genes. Here, we report for the first time *de novo* transcriptome sequencing for *L. olgensis* Henry in order to obtain a comprehensive understanding of the role of NO in plant defense. Several putative metabolic pathways and functions associated with the unique sequences were identified. Moreover, NO has a significant effect on cell wall biosynthesis, and several genes related to plant pathogen infection were upregulated with SNP treatment (*FLS2*, *WRKY33*, *RPM1*, and *PR1*) when *L. olgensis* seedling were exposed to NO. These findings provide a greater understanding of the role NO plays in *L. olgensis* disease response and provide a substantial contribution to the genomic resources.

## Supplementary Materials

Supplementary materials can be found at http://www.mdpi.com/1422-0067/16/12/26117/s1.
