# Supplementary material for: Transcriptomic Response to Nitric Oxide Treatment in Larix olgensis Henry
Source: Int J Mol Sci. 2015 Dec 2;16(12):28582–97. doi: 10.3390/ijms161226117 (PMC4691064; doi:10.3390/ijms161226117)
Supplement: Supplementary file 1 [file ijms-16-26117-s001.zip › ijms-101635-Supplementary figures.pdf]

Supplementary Materials: Transcriptomic Response to Nitric Oxide Treatment in *Larix olgensis* Henry

Xiaoqing Hu, Jingli Yang and Chenghao Li

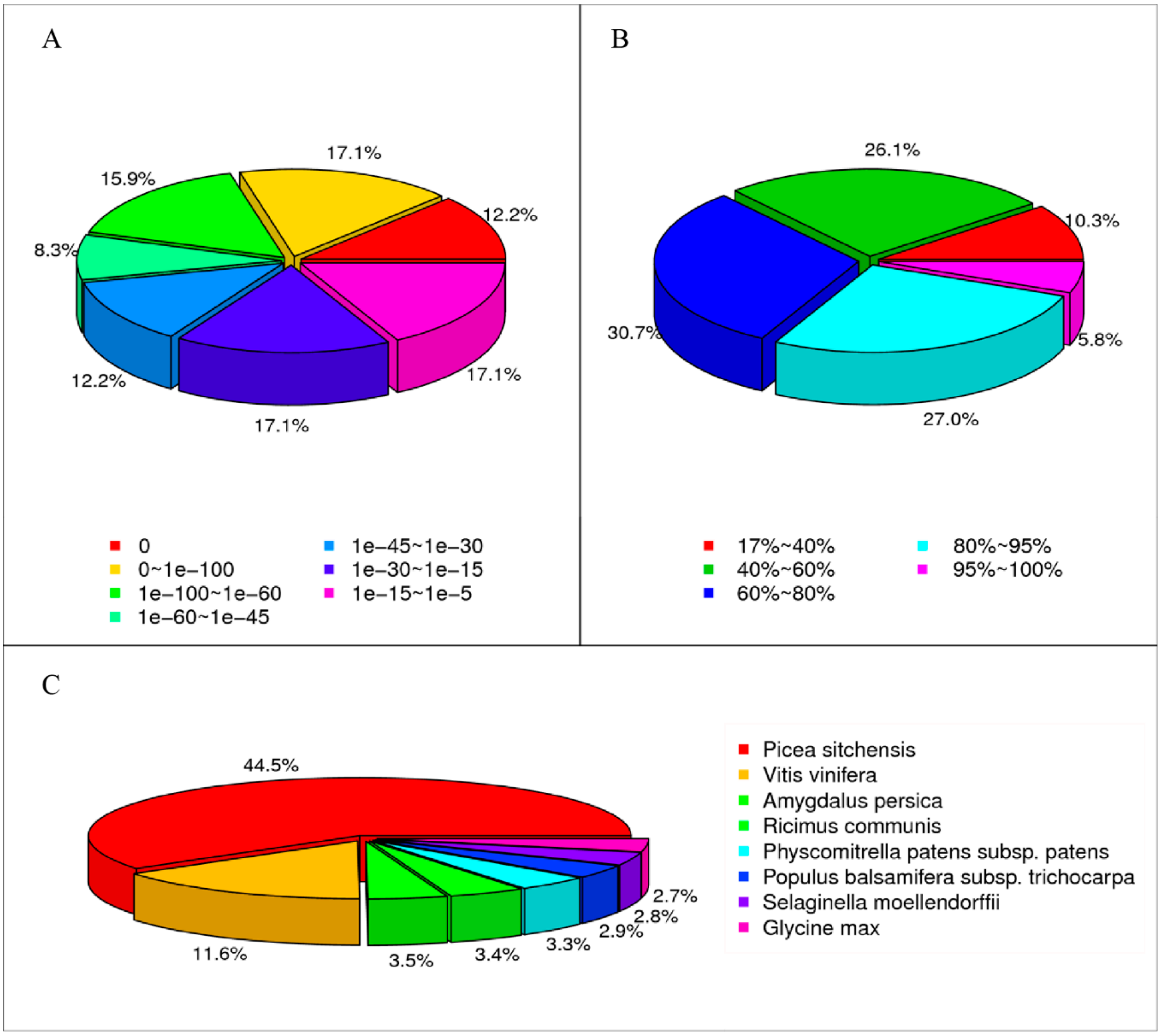

Figure S1. Non redundant (Nr) classification. (**A**) The E-value distribution of the result of Nr annotation; (**B**) The similarity distribution of the result of Nr annotation; (**C**) The species distribution of the result of Nr annotation.

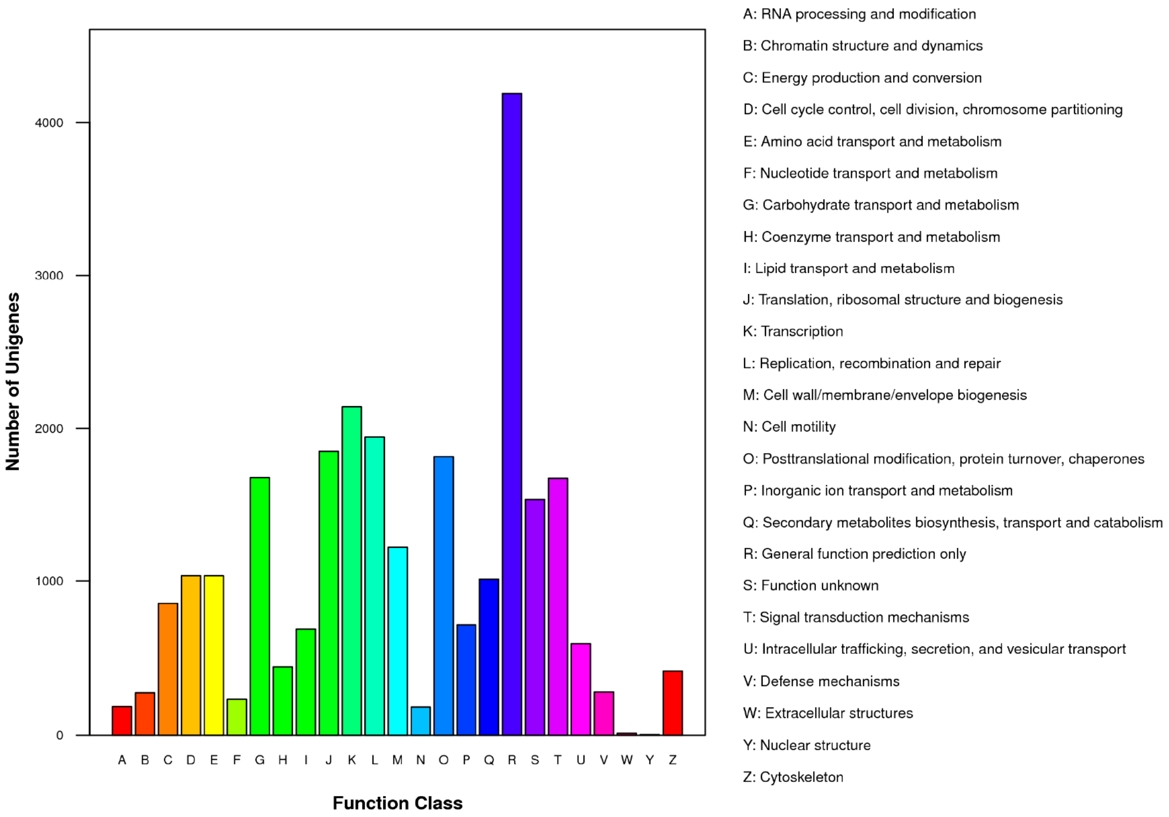

Figure S2. Clusters of orthologous groups (COG) classification. All unigenes were aligned to the COG database to predict and classify possible functions. The horizontal coordinates are COG function classes, and the vertical coordinates are numbers of Unigenes in one class. The notation on the right is the corresponding names from the *X*-axis.

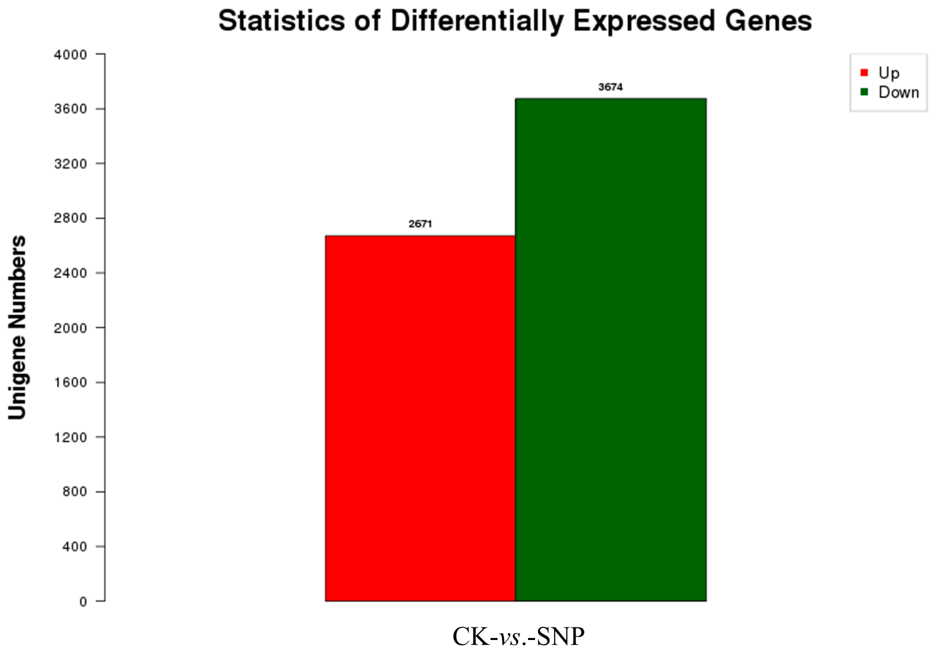

Figure S3. The distribution of differentially expressed genes. The *X*-axis is the control- *vs*.-treatment, and the *Y*-axis is the number of the differentially expressed genes.
